# Characterization of HIV-1 molecular epidemiology and transmitted drug-resistance in newly diagnosed HIV-infected patients in Sichuan, China

**DOI:** 10.1186/s12879-022-07576-z

**Published:** 2022-07-07

**Authors:** Chang Zhou, Shu Liang, Yiping Li, Yan Zhang, Ling Li, Li Ye, Dan Yuan, Ling Su

**Affiliations:** Center for AIDS/STD Control and Prevention, Sichuan Center for Disease Control and Prevention, Chengdu, 610041 Sichuan China

**Keywords:** HIV-1, Genotype, Molecular epidemiology, Transmitted drug resistance

## Abstract

**Background:**

Sichuan province is one of the highest AIDS epidemic provinces in China, with a large number of floating population. The annual number of cases of HIV/AIDS reported in Sichuan has been the highest province in China for several successive years. There is a lack of widespread and representative data on the distribution of HIV genotypes in Sichuan. We aim to investigate the characteristics of HIV-1 molecular epidemiology and transmitted drug-resistance in newly diagnosed HIV-infected patients in Sichuan, China.

**Method:**

Archived plasma samples (n = 1524) from HIV-1 newly-diagnosed individuals in April 2019 were selected by cross-sectional investigation from all 21 cities in Sichuan province. Phylogenetic relationship, transmission cluster, and genotypic drug resistance analyses were performed using HIV-1 polymerase (*pol*) gene sequences. We also analysed the association of demographic and virological factors with transmitted drug-resistance (TDR) and transmission clusters.

**Results:**

Partial *pol* gene sequences were obtained from 1297 cases. HIV-1 epidemic strains in Sichuan province: the majority of genotypes were circulating recombinant form (CRF) 07_BC (675, 52.04%), CRF01_AE (343, 26.45%), CRF08_BC (115, 8.87%), CRF85_BC (67, 5.17%), subtype B (33, 2.54%), the other genotypes only accounted for 4.93%, and unique recombinant forms (URFs) (23, 1.77%) were observed in the study, and the difference of age, ethnicity, education, occupation, region and transmission pathway of different genotypes were statistically significant. According to WHO HIVDR surveillance threshold, the level of TDR has reached a medium level, with 72 of 1297 (5.55%) cases carrying drug-resistance mutation sites, TDR mutation frequency to nonnucleoside reverse transcriptase inhibitors (NNRTIs, 3.85%) was much higher than nucleoside reverse transcriptase inhibitors (NRTIs, 0.31%) and protease inhibitors (PIs, 1.70%), and CRF08_BC was a risk factor for TDR (odds ratio, 8.32; 95% CI 4.38–15.80 for CRF07_BC, P < 0.05). The most common drug resistance HIV-1 mutation pattern for NNRTI was V106 (1.31%, 17/1297) and E138 (1.16%, 15/1297), and for PI was M46 (0.69%, 9/1297). A total of 205 (15.8%) *pol* sequences were involved in the genetic transmission network clusters, CRF01_AE (odds ratio, 2.369; 95% CI 1.659–3.382; *P* < 0.05), subtype B (odds ratio, 13.723; 95% CI 6.338–29.71; *P* < 0.05), drug resistance (odds ratio, 0.306; 95% CI 0.106–0.881; *P* < 0.05) and different levels of education (*P* < 0.05) were significantly associated to be in clusters.

**Conclusion:**

The distribution of HIV-1 genotypes in Sichuan is more diverse and complex, and the Men who have sex with men (MSM) is underrated, arguing for behavior scaling up intervention in this specific population besides the elderly people with heterosexual transmission risk groups. The risk of TDR mutation frequency increased in newly diagnosed patients highlights the significance of genotypic drug resistance monitoring and molecular surveillance of pretreatment HIV-1 drug resistance. The regimen composed of TDF, 3TC and EFV was still currently the preferred solution used free first-line therapy.

## Background

The prevalence of human immunodeficiency virus type 1 (HIV-1) remains a major public health burden in China. Sichuan province is in the interior of Southwest China, adjacent to Yunnan and Guizhou in the South and Tibet in the West. The epidemic of HIV/AIDS has kept rising in Sichuan province since reported the first case of imported AIDS in 1991, the number of newly reported cases per year has increased by about 20% since 2006, by 2019, Sichuan province reported more than 170 thousand cases of HIV/AIDS, with the number of surviving cases ranking first in the country [1].

Among the four phylogeny groups that constitute HIV-1 (M, N, O, P), M group is the virulence factor of AIDS pandemic [2]. There were nine subtypes (A, B, C, D, F, G, H, J and K) and at least 118 circulating recombinant forms (CRFs) (https://www.hiv.lanl.gov/content/sequence/HIV/CRFs/CRFs.html) within HIV-1 group M. With the genetic variation and cross recombination of genes between different genotypes, numerous new circulating recombinant forms (CRFs)/unique recombinant forms (URFs) are emerging, and the global epidemic situations of HIV has changed significantly. HIV enormous genetic variability and rapid evolution have led to its epidemic and therapeutic challenges. Worldwide, the proportion of subtype C was the highest, accounting for 48%, followed by subtype A (12%), subtype B (11%) and CRF02_AG (8%), CRF01_AE (5%), G subtype (5%) and D subtype (2%) [3]. A national study showed that 8 subtypes and 21 CRFs have been identified in China since 2013 [4]. CRF01_AE, CRF07_BC, CRF08_BC, subtype B were the four main subtypes, accounting for 89.0% of all HIV-1 infections [5]. CRF01_AE mainly transmitted by sexual route spread from the southeast coast and southwest border to the whole country [6]. With the wide prevalence of subtypes B and C among IDUs in Yunnan, it was provided sufficient conditions to the hybridization and recombination of HIV-1 strain of and resulting in the formation of recombinant subtypes CRF07_BC and CRF08_BC [7]. Additionally, B subtype mainly originated in Thailand, and this subtype also became the main HIV-1 subtype transmitted by blood in Central China in the 1990s [8].

Since Chinese government officially launched the National Free Antiretroviral Treatment Program (NFATP) in 2003, the number of patients enrolled in this program has increased rapidly year by year, with the increasing coverage of antiviral therapy; the occurrence of drug resistance is also increasing. The emergence of HIV drug resistance (HIVDR) threatens the global scale-up of antiretroviral treatment (ART) for treating HIV infection, which could increase the risk of ART failure. It is important to conduct HIVDR surveys to estimate the transmitted drug-resistance (TDR) rate of ART-naive people living with HIV (PLWH) to provide baseline information for effective ART programs, delaying HIVDR occurrence and developing a rational public health strategy to control HIV prevalence. However, HIVDR testing for ART-naive PLWH is not routinely performed in China as a developing country with limited conditions. The rates of TDR vary throughout the world. In China, the overall prevalence of TDR among ART-naïve individuals in 2004 and 2005 was 3.8% [9], and the rate of TDR was 3.6% in 2015 [10].

Sichuan province has a population of approximately 85 million people, and is a developing area, Sichuan province is the most severely HIV affected area in China [1]. In a previous study, we performed a comprehensive investigation of the HIV epidemic in Sichuan province, 2014 [11] and some cities in Sichuan [12, 13]. With the development of HIV-1 epidemics. With the HIV epidemic growing and treatment scaling up, it is essential to investigate the changing trend of HIV-1 genetics in the province, and to conduct a province-wide transmitted drug resistance (TDR) survey to understand the frequency of transmitted drug-resistant viruses. Therefore, we conducted a large cross-sectional study of the recently infected population identified in April 2019 in the entire Sichuan province. We have used molecular phylogenetic analyses to complement HIV surveillance tasks of Sichuan province. Specifically, we have used this information to infer the distribution and characteristics of HIV-1 subtypes, to analyse which risk groups are currently more vulnerable to TDR HIV infection and in molecular transmitted cluster. The results obtained from this work may be useful in establishing and reinforcing preventive measures in specific target groups.

## Material and methods

### Study participants

A total of 1524 plasma samples were obtained from individuals newly diagnosed as HIV-1 infected patients in April 2019 were selected by cross-sectional investigation from all 21 cities in Sichuan province. The epidemiological data of demographic characteristics (i.e., ethnic, sex, age, and marital status) were acquired from China Information System for Disease Control and Prevention. After eliminating duplicate samples, *pol* sequence (covering 1, 060 base pairs, HXB2: 2, 254–3, 313) information was successfully exported from 1297 PLWH in this study.

### RNA extraction, amplification, and sequencing

About 5 ml of venous blood was extracted. The EDTA-K2 anticoagulated plasma samples were isolated from each participant and preserved in a – 80 °C freezer before sending the cold chain to the Sichuan CDC.

The viral nucleic acid was obtained from 200 μl plasma of PLWH by extraction machines (MagNA Pure LC system, Roche, Branchburg, NJ). Sequences were generated from the HIV-1 *pol*. The Reverse Transcription-Polymerase Chain Reaction (RT-PCR) was used to amplify the full-length protease gene in the *pol* region and the first 300 codons of the reverse transcriptase gene. The PCR products were dealt with electrophoresis with 1% agarose gel, and the amplified positive products were purified and sequenced by Beijing Genomics Research Center Ltd. The detailed amplification and sequencing performed as previously described [14].

### Sequence analysis and genotype determination

The obtained sequence was spliced by using the analysis software Sequencher 5.1. The BioEdit Sequence Alignment Editor was used to edit and correct the sequence. MEGA 7.0 software was used to determine the genotype. All assembled sequences were aligned together with the reference sequences (from the HIV sequence database of Los Alamos National Laboratory in the United States) using Clustal W program in MEGA 7.0, and then further checked manually in Bioedit [15]. FastTree was used to estimate an approximately maximum likelihood phylogenetic tree for *pol* sequences using the GTR + G + I nucleotide substitution model [16], using the ShimodairaHasegawa (SH) test embedded in the software to calculated the node (branch point) value of the evolutionary tree, defined clades with SH-like support ≥ 0.70 [17, 18]. The genotype were preliminarily determined by clustering the sample sequences with international reference strains, then the online analysis tool HIV Databases BLAST (https://www.hiv.lanl.gov/content/index) was used to review the results. Presented phylogenetic tree by using FigTree v1.4.3 (http://tree.bio.ed.ac.uk/software/figtree/). If the sequence whose genotype cannot be confirmed by phylogenetic tree and HIV Databases BLAST were considered as URFs. For example, the sequence between the CRF_01AE and B subtype reference strains in the phylogenetic tree, and the similar sequences in HIV Databases BLAST were 01B genotype, we judged this URF as URF_01B.

### Transmission cluster identification

The gene distance between the clusters was calculated using the software hyphy 2.2.4 in TN93 model, and the relationship between the two sequences was determined by the minimum gene distance method. The transmission network was constructed with a threshold of pairwise genetic distance of 0.5%, which is more appropriate for identifying rapidly growing clusters, and was based on study findings showing that within monoinfected individuals *pol* sequences do not diverge more than 1% during the first 3 years of infection [19]. The molecular transmission clusters were deduced by cytoscape 3.7.2 [20].

### Drug resistance mutation analysis

Drug-resistance were analyzed based on genotypic interpretations defined by the Surveillance Drug Resistance Mutation list recommended by the World Health Organization. The Stanford University HIV DRUG RESISTANCE DATABASE (https://hivdb.stanford.edu/) was used to screen the *pol* sequences for surveillance drug resistance mutations (DRMs), it was evaluated relevant resistance for nucleoside reverse transcriptase inhibitors (NRTIs), nonnucleoside reverse transcriptase inhibitors (NNRTIs), and protease inhibitors (PIs) [21, 22]. If HIV-1 strains carrying at least one TDR mutation were defined as resistant.

### Statistical analysis

Data were entered in a spreadsheet (Micorsoft Office Excel 2013) and analyzed using SPSS 25 software (IBM, Chicago, IL, USA). The potential demographic characteristics (sex, age, ethnicity, marital status, and transmission route) were expressed in percentage, differences between the variables group for genotypes were evaluated using the χ^2^ test. Univariate logistic regression model was used to test the independent variables (i.e. sociodemographic) and transmission drug resistance individuals (1 = drug-resistance, 0 = non drug-resistance), multivariate logistic regression model was used to further test the significance of all micro significant (*P* < 0.1), variables in univariate logistic regression model. Potential demographic differences between patients in cluster and those out of cluster were evaluated by uni-multivariate logistic regression analysis. P value < 0.05 was considered statistically significant.

## Results

### Demographic features and distribution of HIV-1 genetic forms

**Fig. 1:**
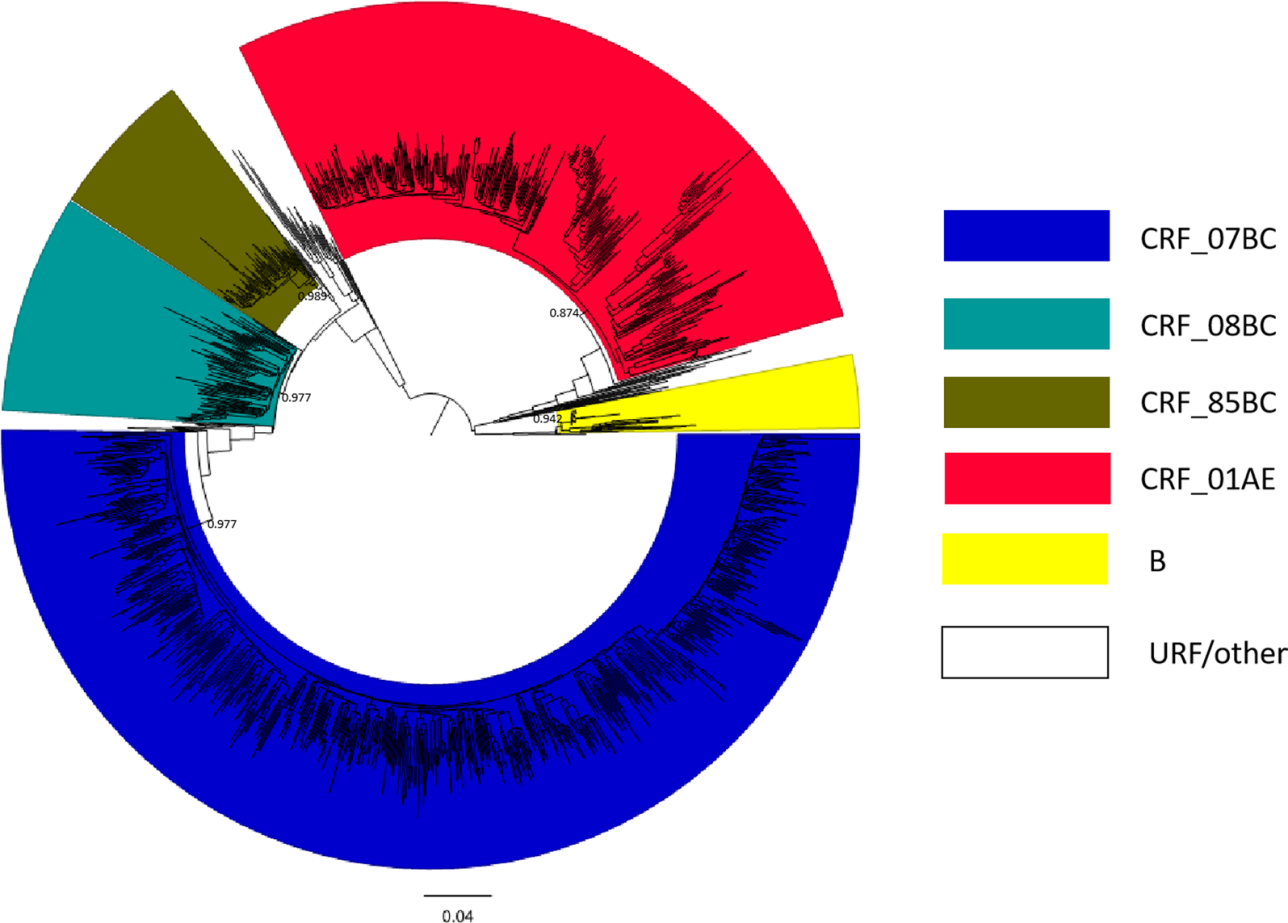
The phylogenetic tree of HIV-1 *pol* sequences obtained from Sichuan. The maximum-likelihood tree obtained using 1062 bp corresponded to the protease region and part of the reverse transcriptase. A total of 675 HIV-1 sequences branched with CRF07_BC reference sequences (depicted in blue). 342 HIV-1 query sequences branched with CRF01_AE reference sequences (depicted in red). 115 HIV-1 CRF08_BC query sequences were identified (depicted in light blue). 67 HIV-1 CRF85_BC query sequences were identified (depicted in brown). 36 query sequences clustered with HIV-1 subtype B reference sequences (depicted in yellow). Other subtypes and URFs were not depicted color. The reference sequences from the Los Alamos HIV sequence database (http://hiv-web.lanl.gov/content/index)

1297 individuals with successfully obtained *pol* sequences from 1524 infected patients with HIV-1, 15 genotypes were identified (Fig. 1). In the phylogenetic tree, sequences belonging to different genotypes clustered separately. Furthermore, several clusters supported by high bootstrap values were also found among different genotypes, suggesting that various founder viruses were introduced into populations separately. Major HIV-1 epidemic strains in Sichuan province were: CRF07_BC (675, 52.04%), CRF01_AE (343, 26.45%), CRF08_BC (115, 8.87%), CRF85_BC (67, 5.17%), subtype B (33, 2.54%). The other genotypes only accounted for 4.93%, of which CRF55_01B (13, 1.00%), CRF105_0108 (11, 0.85%), CRF79_0107 (5, 0.39%), CRF59_01B (3, 0.23%), CRF86_BC (2, 0.15%), CRF57_BC (2, 0.15%), subtype C (2, 0.15%), CRF65_cpx (1, 0.08%), CRF88_BC (1, 0.08%), subtype D (1, 0.08%), and 23 URFs that could not be classified (1.77%). URF including URF_BC (10, 0.77%), URF_01/BC (7, 0.54%), URF_B (4, 0.31%), URF_01/B (2, 0.15%).

**Fig. 2:**
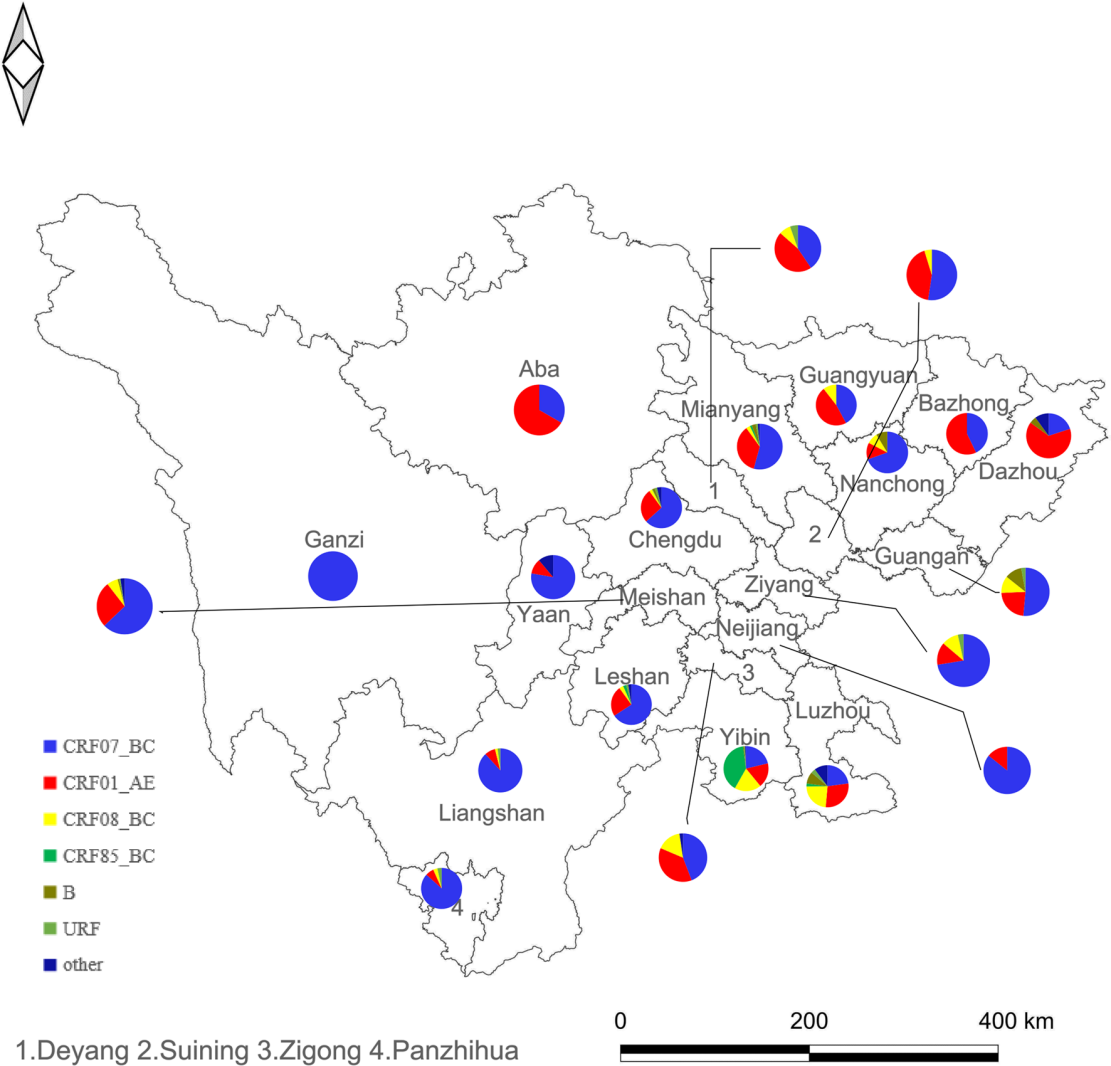
Distribution of HIV-1 genotypes in different cities of Sichuan

It can be seen in Fig. 2 that the genotypes in the central Sichuan Basin (Chengdu plain) are more abundant, the CRF07_BC (86/102, 84.31%) in the Western Sichuan Basin account for a higher proportion, and the CRF01_AE (26/46, 56.52%) in the Northeast Sichuan (Bazhong, Dazhou and Guangyuan) Basin account for more. CRF07_BC in Panzhihua-Xichang region accounts for 87.65% (71/81); most of the CRF85_BC was distributed in southern Sichuan (i.e., Yibin, 57/146, 39.04%); CRF08_BC is mainly distributed in Luzhou and Yibin (60%, 69/115); CRF07_BC (40/175, 22.29%), CRF08_BC (50/175, 28.57%), CRF01_AE (41/175, 23.43%) have similar composition ratios in Luzhou city, and CRF105_0108 of aggregation were found in there.

Demographic information is summarized in Table 1, among the six major genotypes, the difference of age, ethnicity, education, occupation, region and transmission pathway of different genotypes were statistically significant (*P* < 0.05). The majority groups of CRF85_BC (95.53%, 64/67) and CRF105_0108 (90.90%, 10/11) with age ≥ 50 years old were found in the heterosexual transmission; 89.39% of ethnic Yi infected with CRF07_BC; 49.75% of ethnic Han was infected with CRF07_BC, which was found their infection rate was higher than that of other age groups in the 25 year old (48/71, 67.60%) and 25–40 years old (129/183, 70.49%) age group. Among the transmission route of IDUs, mother to child transmission (MCT), and MSM, CRF07_BC was the dominant strain and the composition ratio of was 100%, 77.78% and 54.43%. Furthermore, 13/13 CRF55_01B, 5/5 CRF79_0107, 2/3 CRF59_01B was detected in men.

**Table 1:** General distribution characteristics of epidemic strains in Sichuan province

Variables		Total [%]^a^	The frequency and prevalence of different HIV-1 genotypes (%)^b^												χ^2^		*P*-values	
		n = 1297	CRF07_BC		CRF01_AE		CRF08_BC		CRF85_BC		B		Others^c^					
Gender																		
Male		936 [72.17]	485 (51.81)		246 (26.28)		81 (8.65)		48 (5.12)		20 (2.13)		56 (5.98)		3.866		0.569	
Female		361 [27.83]	187 (51.8)		95 (10.14)		33 (3.52)		18 (1.92)		13 (1.38)		15 (1.6)					
Age (years)																		
< 25		71 [5.47]	48 (67.6)		17 (1.81)		0 (0)		0 (0)		0 (0)		6 (0.64)		83.627		< 0.0001*	
25~		183 [14.11]	128 (69.94)		29 (3.09)		7 (0.74)		0 (0)		6 (0.64)		13 (1.38)					
40~		197 [15.19]	110 (55.83)		45 (4.8)		27 (2.88)		3 (0.32)		4 (0.42)		8 (0.85)					
≥ 50		846 [65.23]	386 (45.62)		250 (26.7)		80 (8.54)		63 (6.73)		23 (2.45)		44 (4.7)					
Ethnicity																		
Han	1212 [93.45]			603 (49.75)		331 (35.36)		111 (11.85)		66 (7.05)		32 (3.41)		69 (7.37)		45.287		< 0.0001*
Yi	66 [5.09]			59 (89.39)		4 (0.42)		1 (0.1)		0 (0)		1 (0.1)		1 (0.1)				
Others	19 [1.46]			10 (52.63)		6 (0.64)		2 (0.21)		0 (0)		0 (0)		1 (0.1)				
Education level																		
Junior college or above		83 [6.40]	49 (59.03)		26 (2.77)		1 (0.1)		0 (0)		1 (0.1)		6 (0.64)		42.786		< 0.0001*	
High school or technical secondary school		99 [7.63]	60 (60.6)		23 (2.45)		3 (0.32)		1 (0.1)		1 (0.1)		11 (1.17)					
Middle school		336 [25.91]	193 (57.44)		73 (7.79)		32 (3.41)		15 (1.6)		7 (0.74)		16 (1.7)					
Primary school or illiterate		779 [60.06]	370 (47.49)		219 (23.39)		78 (8.33)		50 (5.34)		24 (2.56)		38 (4.05)					
Marital status																		
Unmarried		228 [17.58]	140 (61.4)		54 (5.76)		12 (1.28)		4 (0.42)		3 (0.32)		15 (1.6)		22.134		0.104	
Married		727 [56.05]	366 (50.34)		196 (20.94)		67 (7.15)		41 (4.38)		19 (2.02)		38 (4.05)					
Divorced/widowed		337 [25.98]	163 (48.36)		90 (9.61)		35 (3.73)		21 (2.24)		11 (1.17)		17 (1.81)					
Unknown^d^		5 [0.39]	3 (60)		1 (0.1)		0 (0)		0 (0)		0 (0)		1 (0.1)					
Occupation																		
Farmers		843 [65.00]	422 (50.05)		240 (25.64)		83 (8.86)		38 (4.05)		19 (2.02)		41 (4.38)		36.603		0.001*	
Unemployed		184 [14.19]	89 (48.36)		45 (4.8)		15 (1.6)		18 (1.92)		7 (0.74)		10 (1.06)					
Retired		49 [3.78]	30 (61.22)		6 (0.64)		3 (0.32)		6 (0.64)		1 (0.1)		3 (0.32)					
Others/unknown		221 [17.04]	131 (59.27)		50 (5.34)		13 (1.38)		4 (0.42)		6 (0.64)		17 (1.81)					
Route of transmission																		
HET-nonmarital and noncommercial		462 [35.62]	259 (56.06)		120 (12.82)		33 (3.52)		19 (2.02)		11 (1.17)		20 (2.13)		67.382		0.001*	
HET-commercial		556 [42.87]	257 (46.22)		149 (15.91)		62 (6.62)		44 (4.7)		12 (1.28)		32 (3.41)					
HET-nonmarriage		43 [3.32]	21 (48.83)		13 (1.38)		5 (0.53)		1 (0.1)		3 (0.32)		0 (0)					
HET-Spouses		129 [9.95]	72 (55.81)		32 (3.41)		12 (1.28)		2 (0.21)		5 (0.53)		6 (0.64)					
MSM		79 [6.09]	43 (54.43)		23 (2.45)		1 (0.1)		0 (0)		1 (0.1)		11 (1.17)					
IDUs		8 [0.62]	8 (100)		0 (0)		0 (0)		0 (0)		0 (0)		0 (0)					
Mother to child transmission		9 [0.69]	7 (77.77)		1 (0.1)		0 (0)		0 (0)		0 (0)		1 (0.1)					
Others/unknown		11 [0.85]	5 (45.45)		3 (0.32)		1 (0.1)		0 (0)		1 (0.1)		1 (0.1)					
Region^e^																		
Chengdu Plain		729 [56.21]	451 (61.86)		203 (21.68)		29 (3.09)		5 (0.53)		9 (0.96)		32 (3.41)		5188		< 0.0001*	
South Sichuan		371 [28.60]	96 (25.87)		93 (9.93)		76 (8.11)		62 (6.62)		17 (1.81)		27 (2.88)					
Northeast Sichuan		104 [8.02]	49 (47.11)		37 (3.95)		8 (0.85)		0 (0)		7 (0.74)		3 (0.32)					
Panzhihua-Xichang region		81 [6.24]	71 (87.65)		6 (0.64)		2 (0.21)		0 (0)		0 (0)		2 (0.21)					
Northwest Sichuan		12 [0.93]	8 (66.66)		4 (0.42)		0 (0)		0 (0)		0 (0)		0 (0)					

^a^Numbers in square brackets show the proportion of the cases as a percentage of the total 1297 subjects

^b^Numbers in parentheses show the proportion of HIV-1 subtypes as a percentage of each variable

^c^Other subtypes including CRF55_01B (1.00%), CRF105_0108 (0.85%), CRF79_0107 (0.39%), CRF59_01B (0.23%), CRF86_BC (0.15%), CRF57_BC(0.15%), subtype C (0.15%), CRF65_cpx (0.08%), CRF88_BC (0.08%), subtype D (0.08%), and URFs (1.77%)

^d^Set as missing value

^e^Regions are divided according to different geomorphic and cultural characteristics. Chengdu Plain includes cities of Chengdu, Deyang, Leshan, Suining, Ziyang, Mianyang, Yaan and Meishan; South Sichuan includes cities of Yibin, Zigong, Neijiang and Luzhou; Northeast Sichuan includes cities of Bazhong, Nanchong, Dazhou, Guangyuan and Guangan; Panzhihua-Xichang region includes cities of Panzhihua and Liangshan; Northwest Sichuan includes cities of Aba ad Ganzi

^*^P values < 0.05 were considered statistically significant

### Characteristics of transmitted drug-resistant

**Fig. 3:**
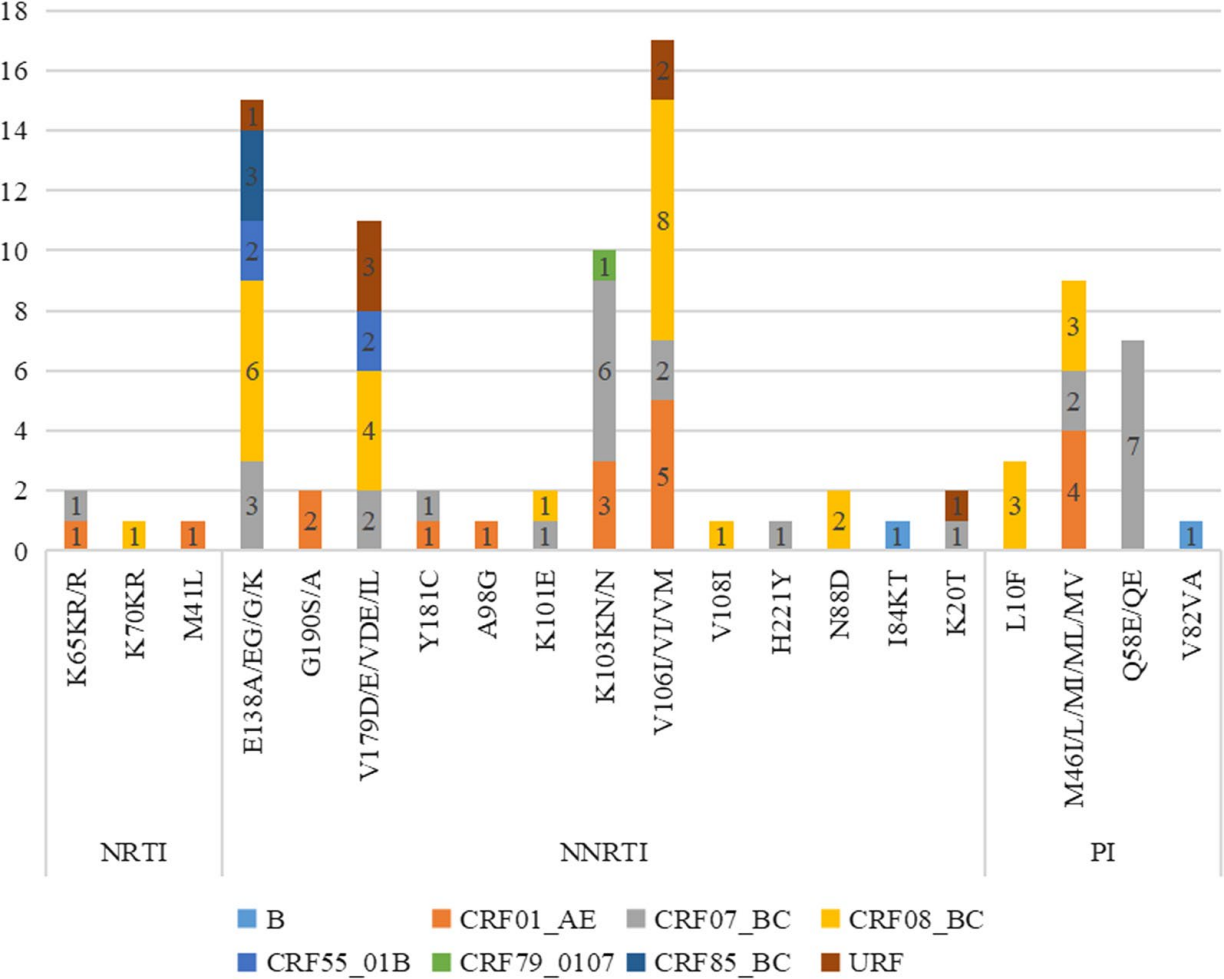
Transmitted drug resistance mutations sites in treatment-naive patients

Of the 1297 subjects, 72 had drug-resistant mutations, among which 12 had high drug resistance, 21 had moderate drug resistance and 55 had low drug resistance, potential drug resistance were 66 cases. 50 cases (3.85%, 50/1297) were found to have non-nucleoside reverse transcriptase inhibitor (NNRTI) resistance mutation, the mutation probability of E138 (30.0%, 15/50), K103 (20.0%, 10/50) and V106 (34.0%, 17/50) was significantly higher than that of other sites. The mutation probability of drug resistance site in CRF07_BC, CRF08_BC, CRF01_AE was higher than that in other genotype, which were resistant to all NNRTIs in varying degrees, and the high resistance was mostly caused by K103N mutation. Four cases (0.31%, 4/1297) had NRTI resistance mutation, the mutation rate was relatively low, but d4T, ddI and TDF caused by K65KR/R mutation were highly resistant. 22 subjects (1.70%, 22/1297) had protease inhibitor (PI) resistance mutation, M46 (36%, 9/22) and Q58 (28%, 7/22) had the highest mutation probability, most of them were low-grade resistance. Additionally, the most frequent mutation was V106, which was observed in 23.6% (17/72) of patients, HIV-1 strains with this mutation exhibited the degree of potential low-level resistance, except for V106VIM resulted in NVF and EFV were highly resistant and dot was moderately resistant. Followed by E138 (20.8%, 15/72), V179 (15.3%, 11/72) and K103 (13.9%, 10/72), all of them were NNRTI related mutations. Moreover, the most resistant drugs were RPV (30.5%, 22/72), NVP (29.2%, 21/72), DOR (25.0%, 18/72). The distribution of drug resistance and mutation sites was shown in Fig. 3.

**Fig. 4:**
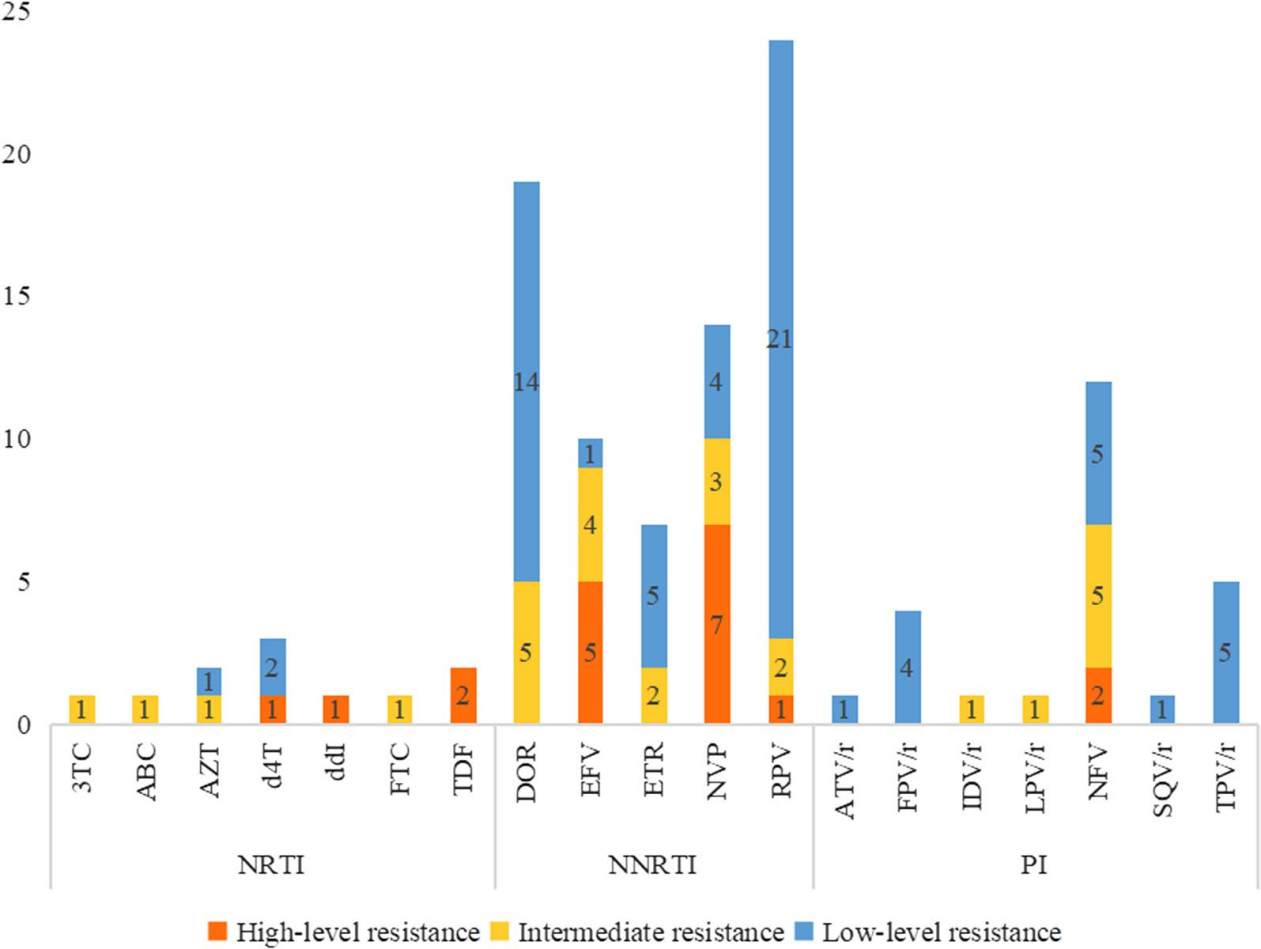
The resistance of HIV-1 to antiretroviral drugs. 3TC: lamivudine; ABC: abacavir; AZT: zidovudine; d4T: stavudine; ddI: didanosine; FTC: emtricitabine; TDF: tenofovir disoproxil fumarate; EFV: efavirenz; DOR: doravirine; NVP: nevirapine; RPV: Rilpivirine; ETR: etravirine; ATV/r: atazanavir/ritonavir; FPV/r: Fosamprenavir/ritonavir; IDV/r: indinavir/ritonavir; LPV/r: lopinavir/ritonavir, NFV: nelfinavir, SQV/r: saquinavir/ritonavir; TPV/r: tipranavir/ritonavir

**Fig. 5:**
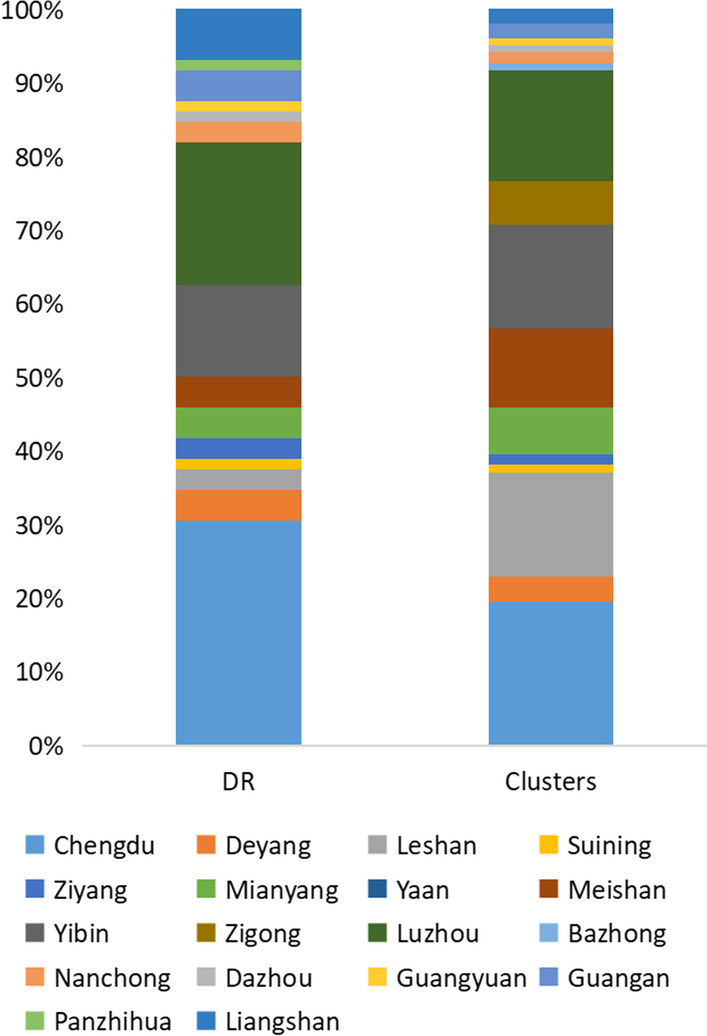
TDR and clustering rate of different cities in Sichuan

The drug resistance level is shown in Fig. 4. The level of HIV-transmitted drug resistance is 5.55% (72/1297) in Sichuan province. The level of transmissible drug-resistance in city of Liangshan, Luzhou, Deyang and Guang’an the high proportion, were 9.80%, 8.00%, 8.11% and 8.57% (Fig. 5). The overall prevalence of HIV-1 TDR to NNRTI (3.85%, 50/1297) was higher than that to NRTI (0.31%, 4/1297) and PI (1.70%, 22/1297). The majority (95.8%, 69/72) of HIV-1 drug-resistance variants displayed a single drug class resistance mutation, three cases (0.23%) contained mutations associated with drug resistance to both PIs and NNRTIs. One case (0.08%) contained mutations conferred to NRTIs sand NNRTIs. No HIV-1 strain with TDR mutations to triple classes of drugs was found in this study.

### Analysis of risk factors of transmitted drug resistance

Taking drug resistance as an independent variable, 8 variables including gender, age at the time of HIV diagnosis, nationality, education level, marital status, occupation, route of infection and genotype as dependent variables, logistic stepwise regression model were used to analyze the risk factors of TDR (Table 2). The results showed that the HIV-1 genotype was a potential influencing factor associated with TDR. CRF07_BC is one of the three main epidemic strains in Sichuan province, which the resistance rate was 3.41% (23/675), the drug resistance rate of CRF01_AE was 4.08% (14/343), CRF08_BC (87%, 24/115) had a higher risk of TDR (odds ratio, 8.32; 95% CI 4.38–15.80; *P* < 0.05), while other variables had no significant effect on drug resistance (*P* > 0.05).

**Table 2:** Demographic characteristics and factors associated with drug resistance

Variables	DR (%)^a^	Non-DR (%)^a^	*OR *(95% *CI*)	*P*-values	*AOR *(95% *CI*)	*P*-values
	*n* = 138	*n* = 1159				
Age (years)						
< 25^b^	6 (8.45)	65 (91.54)	1			
25~	13 (7.1)	170 (92.89)	1.42 (0.38–5.33)	0.608		
40~	13 (6.59)	184 (93.4)	0.99 (0.21–4.57)	0.987		
≥ 50	40 (4.72)	806 (95.27)	0.80 (0.17–3.71)	0.776		
Gender						
Male^b^	51 (5.44)	885 (94.55)	1			
Female	21 (5.81)	340 (94.18)	1.05 (0.59–1.88)	0.863		
Ethnicity						
Han^b^	66 (5.44)	1146 (94.55)	1			
Yi	5 (7.57)	61 (92.42)	0.78 (0.17–3.52)	0.75		
Others	1 (5.26)	18 (94.73)	0.96 (0.09–6.49)	0.803		
Marital status						
Unmarried^b^	16 (7.01)	212 (92.98)	1			
Married or living	36 (4.95)	691 (95.04)	0.86 (0.38–1.95)	0.72		
With spouse						
Divorced/widowed	20 (5.93)	317 (94.06)	1.08 (0.45–2.60)	0.856		
Others^c^	0 (0)	5 (100)	–	–		
Education level						
Primary school and below^b^	43 (5.51)	736 (94.48)	1			
Middle school	19 (5.65)	317 (94.34)	0.93 (0.48–1.79)	0.823		
High school or technical secondary school	5 (5.05)	94 (94.94)	0.61 (0.19–1.97)	0.41		
Junior college or above	5 (6.02)	78 (93.97)	0.73 (0.19–2.86)	0.656		
Occupation						
Farmers^b^	39 (4.62)	804 (95.37)	1			
Unemployed	15 (8.37)	164 (91.62)	1.87 (0.91–3.82)	0.087		
Retired	1 (2.04)	48 (97.95)	0.51 (0.06–4.12)	0.532		
Others	17 (7.52)	209 (92.47)	1.58 (0.72–3.45)	0.25		
Route of transmission						
HET^b^	60 (5.04)	1130 (94.95)	1		1	
MSM	6 (7.59)	73 (92.4)	1.34 (0.42–4.23)	0.622	1.91 (0.77–4.77)	0.163
IDUs	1 (12.5)	7 (87.5)	4.63 (0.46–46.69)	0.193	4.86 (0.57–41.44)	0.148
Others	5 (25)	15 (75)	7.55 (1.81–31.43)	0.005	8.13 (2.70–24.53)	< 0.001
Genotype						
CRF07_BC^b^	23 (3.42)	649 (96.57)	1		1	
CRF01_AE	14 (4.1)	327 (95.89)	1.39 (0.68–2.83)	0.362	1.29 (0.65–2.57)	0.465
CRF08_BC	24 (20.86)	91 (79.13)	8.67 (4.43–16.96)	< 0.001*	8.32 (4.38–15.80)	< 0.001*
CRF85_BC	3 (4.54)	63 (95.45)	1.70 (0.47–6.15)	0.42	1.62 (0.47–5.61)	0.447
B	1 (3.03)	32 (96.96)	0.72 (0.08–6.10)	0.759	0.88 (0.11–6.88)	0.901
Others	7 (10)	63 (90)	3.73 (1.53–9.07)	0.004	3.43 (1.44–8.20)	0.005

^a^DR indicates individuals with drug-resistant mutations, non-DR indicates individuals without drug-resistant mutations, and numbers in parentheses show the proportion for each variable

^b^The reference value of this subgroup of variables

^c^Set as missing value

^*^P values < 0.05 were considered statistically significant

### Transmission clusters

Transmission cluster analysis identified 76 clusters containing 205 sequences (205/1297, 15.8%), the cluster sizes ranged between 2 and 24, there were 72 (94.7%) clusters with size < 5, and 4 (5.3%) clusters with size ≥ 5. With regard to the characteristics of individuals involved in clusters in comparison with those out of clusters, CRF01_AE (odds ratio, 2.369; 95% CI 1.659–3.382; *P* < 0.05), subtype B (odds ratio, 13.723; 95% CI 6.338–29.71; *P* < 0.05), drug resistance(odds ratio, 0.306; 95% CI 0.106–0.881; *P* < 0.05) and different levels of education(*P* < 0.05) were significantly associated to be in clusters (Table 3). We also observed that only 4 drug-resistance cases were included in the transmission cluster, and which were concentrated in two clusters that contained only two individuals. It was worth noting that the clustering rate of Leshan, Zigong and Bazhong were much higher than that of other cities (Fig. 5). The largest cluster was composed of individuals infected with subtype B (n = 24), and Luzhou accounted for the majority of cases (15/24, 62.5%) in the largest molecular cluster, but, most clusters were composed of CRF07_BC and CRF01_AE cases. The other three relatively large clusters were two CRF01_AE and one CRF85_BC. We analyzed the infection routes and found that 98.5% of the putative transmission cluster might occurred in heterosexual individuals, among them 101 (49.3%) cases said they have commercial sexual.

**Table 3:** Molecular transmission cluster characteristics of Sichuan patients

Variables	Total [n = 1297]^a^	In cluster(n = 205; 15.81%)	Out of cluster (n = 1092; 84.19%)	OR (95% *CI*)	*P*-values	*AOR *(95% *CI*)	*P*-values
Gender							
Male^b^	936 [72.17]	148 (15.81)	788 (84.18)	1			
Female	361 [27.83]	57 (15.79)	304 (84.21)	0.857 (0.592–1.24)	0.413		
Age (years)							
< 25^b^	71 [5.47]	2 (2.82)	69 (97.18)	1			
25~	183 [14.11]	14 (7.65)	169 (92.34)	1.434 (0.283–7.271)	0.664		
40~	197 [15.19]	25 (12.69)	172 (87.3)	1.406 (0.267–7.412)	0.688		
≥ 50	846 [65.23]	164 (19.39)	682 (80.61)	1.875 (0.36–9.777)	0.456		
Ethnicity							
Han^b^	1212 [93.45]	200 (16.5)	1012 (83.49)	1			
Yi	66 [5.09]	5 (7.58)	61 (92.42)	0.669 (0.22–2.038)	0.48		
Others	19 [1.46]	0 (0)	19 (100)	-	0.998		
Marital status							
Unmarried^b^	228 [17.58]	22 (9.64)	206 (90.35)	1			
Married	727 [56.05]	133 (18.29)	594 (81.7)	1.149 (0.668–1.977)	0.616		
Divorced/widowed	337 [25.98]	49 (14.54)	288 (85.45)	0.75 (0.412–1.366)	0.347		
Unknown^c^	5 [0.39]	1 (20)	4 (80)	–	–		
Occupation							
Farmers^b^	843 [65.00]	159 (18.86)	684 (81.13)	1			
Unemployed	184 [14.19]	22 (12.29)	157 (87.7)	0.851 (0.495–1.462)	0.559		
Retired	49 [3.78]	4 (8.16)	45 (91.83)	0.456 (0.151–1.375)	0.163		
Others/unknown	221 [17.04]	20 (8.84)	206 (91.15)	0.749 (0.417–1.345)	0.334		
Route of transmission							
HET^b^	1190 [91.75]	202 (16.97)	988 (83.02)	1	0.429		
MSM	79 [6.09]	1 (1.26)	78 (98.73)	0.175 (0.022–1.387)	0.099		
IDUs	8 [0.62]	0 (0)	8 (100)	–	0.999		
Others/unknown	20 [1.54]	2 (10)	18 (90)	0.8 (0.139–4.594)	0.802		
Drug resistance^d^							
DR^b^	72 [5.55]	4 (5.55)	68 (94.44)	1		1	
Non-DR	1225 [94.45]	201 (16.4)	1024 (83.59)	0.379 (0.132–1.092)	0.072	0.306 (0.106–0.881)	0.028*
Subtype							
CRF07_BC^b^	675 [52.04]	73 (10.81)	602 (89.18)	1		1	
CRF01_AE	343 [26.45]	78 (22.74)	265 (77.25)	2.161 (1.497–3.119)	< 0.001*	2.369 (1.659–3.382)	< 0.001*
CRF08_BC	115 [8.87]	15 (13.04)	100 (86.95)	1.158 (0.624–2.15)	0.641	1.26 (0.685–2.314)	0.457
CRF85_BC	67 [5.17]	13 (19.4)	54 (80.59)	1.546 (0.785–3.044)	0.208	1.686 (0.873–3.258)	0.120
B	33 [2.54]	21 (63.63)	12 (36.36)	14.692 (6.607–32.668)	< 0.001*	13.723 (6.338–29.71)	< 0.001*
Others	64 [4.93]	5 (7.81)	59 (92.18)	0.716 (0.272–1.885)	0.499	0.761 (0.293–1.977)	0.576
Education level							
Primary school or illiterate^b^	779 [60.06]	155 (19.89)	624 (80.1)	1		1	
Middle school	336 [25.91]	40 (11.9)	296 (88.09)	0.651 (0.427–0.993)	0.046*	0.584 (0.396–0.86)	0.006*
High school or technical secondary school	99 [7.63]	9 (9.09)	90 (90.9)	0.707 (0.305–1.643)	0.421	0.457 (0.221–0.943)	0.034*
Junior college or above	83 [6.40]	1 (1.20)	82 (98.80)	0.129 (0.015–1.101)	0.061	0.049 (0.007–0.362)	0.003*

^a^Numbers in square brackets show the proportion of the cases as a percentage of the total 1297 subjects

^b^The reference value of this subgroup of variables

^c^Set as missing value

^d^DR indicates individuals with drug-resistant mutations, non-DR indicates individuals without drug-resistant mutations

^*^P values < 0.05 were considered statistically significant

## Discussion

In this study, we conducted a cross-sectional HIV-1 molecular epidemiological study to track the characteristics and distribution of HIV-1 genotypes and TDR in newly diagnosed infections in Sichuan. For the first time, the TDR of HIV-1 infection in Sichuan, which was one of the highest AIDS epidemic provinces, was analyzed.

This results were inconsistent with the national monitoring in 2016 [23] and the survey results in some other areas [24, 25], while were similar to that of the study conducted in Sichuan 2014 [11]. Compared with the 2014 survey, it is found that the PLWH in this study were older and less educated, shown that elderly people with low educational background in our province are the people who need to be paid attention to. Heterosexual transmission is likely to be the main route of infection, in which the proportion of commercial heterosexual is the highest, moreover, transmission cluster analysis showed similar results. It showed that commercial sexual activity is an infection factor worthy to be attached in Sichuan. 67.65% of HIV-infected men were over 50 year-old, who infection came mainly from commercial sexual activity, as such groups often exist with spouse separation and widowhood, their sexual needs are hard to be met, and their cognition of AIDS is insufficient. Note that although men younger than 25 years old account for a low proportion of infected people, most of their infection routes are male transmission, which increases with the decrease in age. There was little difference in transmission routes among women of different ages, mainly noncommercial heterosexual transmission, suggesting that further attention should be paid to the source of infection of such populations for more accurate intervention.

A total of 15 HIV-1 genotypes were detected, and 4 URFs were identified. It showed a much more gene diversity of HIV in Sichuan [11], which may reflect the active mobility of people across the province [26]. Similar to the survey in 2014 [11], the main prevailing HIV-1 genotypes in Sichuan remain CRF07_BC and CRF01_AE, but the proportion decreased slightly, which was obviously different from other regions that CRF01_AE (i.e., Anhui, Liaoning and Guangxi), B (i.e., Henan), CRF08_BC (i.e., Yunnan) was the dominant strain [25, 27–29]. CRF01_AE was identified in the 1990s as being imported from Thailand to Southwestern China (i.e., Yunnan and Guangxi) in commercial sex worker (CSW) [30, 31]. The CRFs of CRF07_BC and CRF08_BC have common origin which were first circulating in intravenous drug users (IDUs) in Yunnan [7, 32], which was introduced from and from Liangshan to Sichuan [33]. The proportion of CRF08_BC and CRF85_BC among HIV-1 PLWH in Sichuan increased from 4.96% and 3.39% to 8.87% and 5.17% in 2019, and eight different genotypes were identified more than in 2014 [11]. The proportion of URFs also increased significantly, which may be due to the complexity of HIV-1 gene pool caused by long-term epidemic. The genetic diversity of HIV-1 is abundant in Sichuan, in addition to the known CRFs, some URFs were also detected in Sichuan [34]. URFs contributed to the formation of novel CRFs, recently, new CRFs were identified in Sichuan [13, 14, 35].

In our study, most of the infected people entering the transmission network presumed be heterosexual transmission, but, research in Guangxi showed that most HIV-1 infection clusters were MSM [36], and Liu suggested that factors such as sex, mode of transmission, education level and ethnicity were not significantly correlated with access to the genetic transmission network in Liangshan [37]. It showed that the situation of transmission networks in different regions was various, and targeted prevention and control measures need to be put forward for different regions, especially in cities with high clustering rate. In addition, we would focus on a high clustering rate of subtype B (66.67%, 24/36), which was much higher than other genotypes (10–25%), it is different from the local epidemic tendency. This result showed that timely genotype monitoring is conducive to more accurate prevention and control of HIV transmission.

The results also showed that there were significant differences in the distribution of genotypes in different regions. The frequent reconstitution of the HIV genome will accelerate the evolution of the HIV, which may lead to the emergence of a highly adaptive virus [38, 39]. It was worth noting that the CRFs in Leshan, Chengdu and Luzhou are more complex. The existence of multiple genotypes increases the probability of mutual recombination to form new CRFs/URFs, therefore, it is necessary to further strengthen the monitoring of HIV genotype in Sichuan province, timely grasp the epidemic trends and reduce the generation of CRFs.

Recently, the trend of virus strain diversification in Sichuan province was gradually obvious, CRF55_01B [40], CRF79_0107 [41], CRF59_01B [42, 43] were found for the first time in China’s MSM population. In this study, these three CRFs were detected in men, only one female was transmitted by spouse, but only 38.46% (5/13) CRF55_01B, 40% (2/5) CRF79_0107 PLWH were transmitted by MSM, and the rest were heterosexual sexual transmission. There may be a concealed sexual orientation because of the social homosexual cultural identity and discrimination [44]. Some MSM will have sex with women inside and outside the marriage, and increase the difficulty of AIDS prevention and treatment [45], and suggest that the proportion of homosexual transmission in this province may be underestimated. CRF105_0108 was the genotype found for the first time among heterosexual people in Liangshan Prefecture, Sichuan province, which was found that there was an aggregated epidemic in Luzhou, and one case is also found in Meishan, suggesting that this CRF may have spread in Sichuan province, and 54.54% (6/11) PLWH infection with this CRF were transmitted by commercial heterosexual, indicating that commercial sex workers (CSWs) need to be further found in Luzhou to reduce virus transmission.

Another serious consequence of the high variability of HIV-1 is drug resistance, which is a new threat to epidemic control and can lead to treatment failure and further transmission of resistant HIV. Our study in Sichuan showed that the overall prevalence of TDR was 5.55% among the 1297 participants, belonging to the moderate drug resistance level (5–15%) according to WHO HIVDR surveillance threshold [46]. The result was higher than the currently reported national total prevalence rate and transmissible drug resistance rate of 3–5% [5, 29, 47, 48]. Su [49] found through meta-analysis that the rate of transmissible drug resistance in Beijing, Henan and Hubei has reached the level of moderate drug resistance. The studies of Zhejiang [50] and Shanghai [51, 52] showed that the rate of transmissible drug resistance has been greater than 10%, which may be due to the early start of antiretroviral treatment in some areas and large treatment coverage. The pretreatment drug resistance rate was 9.9% in Liangshan Prefecture from 2017 to 2018 [53], the results were in keeping with our findings that the drug resistance rate of Liangshan was 9.8%. The high rate of TDR in some cities indicated that the monitoring of TDR rate should be carried out in these cities. It could be seen that the longer the antiviral time, the higher the proportion of drug resistance. With the increase in the number of people receiving antiviral treatment, the risk of HIV drug resistance also increases, which may lead to the increase the transmissible drug resistance rate, it directly affects the effect of antiviral therapy. The drug resistance survey results of infected people receiving antiviral treatment showed that the national acquired drug resistance rate was 8.6% [54], and the drug resistance rate of infected people who fail to inhibit the virus exceeds 50% [55, 56]. TDR was less frequent among individuals involved in clusters (1.95%) compared with those out of clusters (6.23%), which might be explained by several factors. First, we should consider that our analysis is only including sequences of newly-diagnosed individuals, we can’t rule out several infector infected with individuals who failed drug treatment. Secondly, due to the lack of CD4 and new infection detection data of these cases, we cannot judge that all individuals are recently infected. Some drug-resistant individuals may have been infected for a long time, resulting in drug-resistant mutations of virus strains in vivo.

According to our study, TDR mutation frequency to NNRTIs was much higher than NRTIs and PIs, due to NNRTI had a low resistance barrier, and it was more prone to drug resistance [57]. Because of the limited availability of drugs in China, the regimen composed of TDF, 3TC and EFV was currently the most commonly used free first-line therapy. The above three kinds of drugs exhibited the degree of resistance was mainly at a potential low level, therefore, the first-line treatment drugs can still be used continuously in Sichuan province. The most frequent NNRTI-associated DRMs were V106 and E138, which were mainly resulted in low-resistance to RPV and DOR, whereas were K103N in whole country [5, 25] and V179D/E in Shanghai [51]. There were 21 cases of DOR resistance, accounting for a high proportion, DOR is a new NNRTI, which has not provided through the NFATPI in China, suggesting that the use of DOR in the future needs to pay close attention to whether it will have a certain impact on the treatment effect. The proportion of K103N mutation in our study was also high, and it is highly resulted in resistant to EFV and NVP, these two drugs were free NNRTI drugs used in China, therefore, we need to be vigilant about the mutation of K103N. The NRTI-associated DRMs were K65KR/R, K70KR and M41L, among which K65KR/R mutation causes high resistance to d4T, DDI and TDF, but M184V [5], the most common mutation site of NRTI resistance, is not found in our study, which may be related to the current first-line treatment scheme and the low rate of NRTI resistance in Sichuan province. The main DRMs of PI were M46 and Q58, which produced low or intermediate-resistance to NFV, and low-resistance to TPV/r. The most frequent DRMs M46 was consistent in previous reports [5, 48, 51], and the main DRMs Q58 could be related to the high prevalence of CRF07_BC in Sichuan. Thus, it was considered that, the mutation of drug resistance discovered in the study could be induced by the mutation of exogenous population or non-drug selection pressure.

However, our study has limitations. First, a potential sampling bias, on the one hand, in our study only one cross-sectional study was conducted, and the results could not be used to observe the dynamics of the local HIV epidemic, on the other hand, we could analyze only the samples that had been diagnosed, but those that had been infected but not diagnosed could not be included in the analysis. Second, our analysis of TDR concentrated only on NRTI, NNRTI and PI, not containing integrase inhibitors, which are increasingly infected people began using this drug.

## Conclusions

In summary, our study provides a comprehensive molecular epidemiologic dataset to understand the diversity and distribution of HIV genotypes in newly diagnosed HIV-infected patients, and supplements in transmitted drug-resistance in Sichuan province. The distribution of HIV-1 genotypes in Sichuan is more diverse and complex than 2014, with CRF07_BC and CRF01_AE still as the predominant genotypes. The MSM is underrated, arguing for behavior scaling up intervention in this specific population besides the elderly people with heterosexual transmission risk groups. The risk of TDR mutation frequency increased in newly diagnosed patients highlights the significance of genotypic drug resistance monitoring and molecular surveillance of pretreatment HIV-1 drug resistance, the regimen composed of TDF, 3TC and EFV was still currently the preferred solution used free first-line therapy.

## Data Availability

The international reference strain sequences determined in this study are available in the [Los Alamos National Laboratory HIV Database] repository, [www.hiv.lanl.gov]. Other datasets used and/or analyzed during the current study available from the corresponding author on reasonable request.
